# Comparison of robustness to outliers between robust poisson models and log-binomial models when estimating relative risks for common binary outcomes: a simulation study

**DOI:** 10.1186/1471-2288-14-82

**Published:** 2014-06-26

**Authors:** Wansu Chen, Jiaxiao Shi, Lei Qian, Stanley P Azen

**Affiliations:** 1 Kaiser Permanente Southern California, Department of Research and Evaluation, Pasadena, CA, USA; 2 Department of Preventive Medicine, Keck School of Medicine, University of Southern California, Los Angeles, CA, USA

**Keywords:** Relative risk, Risk ratio, Log-binomial regression, Robust poisson regression, Outliers, Common binary outcomes

## Abstract

**Background:**

To estimate relative risks or risk ratios for common binary outcomes, the most popular model-based methods are the robust (also known as modified) Poisson and the log-binomial regression. Of the two methods, it is believed that the log-binomial regression yields more efficient estimators because it is maximum likelihood based, while the robust Poisson model may be less affected by outliers. Evidence to support the robustness of robust Poisson models in comparison with log-binomial models is very limited.

**Methods:**

In this study a simulation was conducted to evaluate the performance of the two methods in several scenarios where outliers existed.

**Results:**

The findings indicate that for data coming from a population where the relationship between the outcome and the covariate was in a simple form (e.g. log-linear), the two models yielded comparable biases and mean square errors. However, if the true relationship contained a higher order term, the robust Poisson models consistently outperformed the log-binomial models even when the level of contamination is low.

**Conclusions:**

The robust Poisson models are more robust (or less sensitive) to outliers compared to the log-binomial models when estimating relative risks or risk ratios for common binary outcomes. Users should be aware of the limitations when choosing appropriate models to estimate relative risks or risk ratios.

## Background

When the outcome of a study is binary, the most common method to estimate the effect is to calculate an odds ratio (OR) as an estimate of relative risk (RR) using a logistic regression. When the outcome prevalence is high (>10%), the OR can still be estimated by using the logistic regression model, but the OR is no longer an acceptable estimate for RR [1]. Interpreting the OR as being equivalent to the RR still occurs in medical research, leading to overstated effect in the study findings [2,3]. The degree of overstatement depends on the outcome rate (e.g., disease prevalence). A higher outcome rate leads to higher degree of exaggeration. Zhang and Yu proposed a formula to convert the adjusted OR derived from the logistic regression model to a risk ratio in studies with common outcomes [4]. However, the method was noted by McNutt et al. to produce biases in both point estimates and confidence intervals (CIs) [5]. Miettinen suggested the doubling-of-cases method to estimate OR as an approximation of RR using logistic regression based on a modified dataset [6]. Schouten et al. improved the method by applying robust standard errors [7]. However, this method was mentioned by Skov et al. to produce prevalence greater than one [8]. Several model-based methods have been proposed to estimate RR and its CI directly [9]. The most popular ones are the robust (also known as modified) Poisson model [10-12] and the log-binomial model [8,11,13]. The performance based on simulations seemed to be equally good between the log-binomial model and the robust Poisson model [3,11,12,14] when sample sizes are reasonably large. Out of the two models, it was reported that the robust Poisson may be less affected by outliers compared to the log-binomial method [15]. However, the research in this area is very limited. The purpose of this study is to evaluate the performance of the two methods using simulation in several scenarios when outliers exist and to provide insight into the selection of the appropriate models.

## Methods

### Estimation methods

The Poisson regression uses a logarithm as the natural link function under the generalized linear model framework. When the outcome is common, the standard Poisson regression over estimates the variance for the measured effect [10-12]. The robust Poisson regression model uses the classical sandwich estimator under the generalized estimation equation (GEE) framework to correct the inflated variance (also known as over-dispersion) in the standard Poisson regression. This correction can be achieved by using the REPEATED statement in SAS Proc GENMOD [12] or the ROBUST option in STATA’s Poisson procedure [11]. The estimators based on the robust Poisson models are pseudo-likelihood estimators.

The log-binomial regression approach models the probability of having the outcome (e.g., disease) based on the binomial distribution and logrithm of the probability as the link function in a generalized linear model [8,13]. The log-binomial model attempts to find a MLE if it exists. MLEs are preferred estimators because they carry many good properties including higher efficiency compared to non-MLE estimators. The analyses can be performed using SAS’s GENMOD, R’s GLM or STATA’s GLM procedure. However, for many situations in which quantitative covariates exist, the MLE can be on the boundary of the parameter space (i.e. the predicted probability of the outcome equals to 1), leading to the difficulty of finding the MLE. To address the non-convergence issue, a SAS macro called “COPY” was developed [16] and later enhanced [17] to increase the chance of finding an approximate MLE inside the interior of the parameter space using PROC GENMOD. The COPY method uses the results of the log-binomial regression if the model converges. When the log-binomial regression fails to converge, the COPY method creates C (usually a large number) copies of the original data and one copy of the original data with the outcome variable reversed (Y converted to 1-Y) and then takes the modified data to generate the estimates [16]. The enhanced version of the COPY method simply generated virtual copies by applying weights of C and 1, respectively, to the original dataset and the dataset with the outcome variable reversed [17]. The choice of C impacts the accuracy and the efficiency of the estimates as well as the model convergence. A larger C produces results that are closer to MLEs yet leads to a higher level of difficulty in finding the MLEs due to failure of convergence [17].

### Simulation methods

#### Design of uncontaminated datasets

Let *X* be a binary treatment/exposure variable (*X* = 1 for treatment/exposure and *X* = 0 for non-treatment/non-exposure) from a binomial distribution with the probability of *X* = 1 fixed at 50%. Let *Y* be a binary common outcome from a population with the probability of *Y* = 1 varying from 10%, 25% to 40%. Let *Z* be a continuous confounder following the Beta (6, 2) distribution. The Beta distribution offers a wide range of possible forms. The parameters chosen (*α = 6* and *β* = 2) provided the distribution with a mean of approximately 0.75. If *Z* × 100 represents the ages of study subjects, they came from an elderly population with the average age being 75 years old. The relationship between *X* and *Z* is defined by the equation logit (*p*_*x*_) = *a*_0_ + *a*_1_*Z*, where *a*_1_ = log(2) or log (4) to indicate moderate or strong association, respectively. The relationship between *Y* and *X* is log-linear with adjusted *RR* being 1.5 or 2. *Z* is designed as a linear or quadratic confounder.

$$\begin{array}{l} \mathrm{Linear}\;\mathrm{cofounder}\;Z:\log\left(p_{v}\right)=\beta_{0}+\beta_{1}X+\beta_{2}Z,\;\mathrm{or}\\ \mathrm{Quadratic}\;\mathrm{cofounder}\;Z:\log\left(p_{v}\right)=\beta_{0}+\beta_{1}X+\beta_{2}Z\\ \;+\left(0.5\times \beta_{2}\right)Z^{2},\;\\ \mathrm{where}\;\beta_{1}=\log\left(1.5\right)\;\mathrm{or}\;\log\left(2\right),\;\mathrm{and}\;\beta_{2}=\log\left(2\right)\;\mathrm{or}\;\log\left(4\right) \end{array}$$

The value of *β*_1_ in the models denotes the effect due to exposure on the log-relative risk scale (i.e. *β*_1_ = log (RR)). To simplify the design, we set *β*_2_ = *α*_1_. There were a total of 24 scenarios (2 RRs, 3 outcomes, 2 levels of association, 2 types of confounders). A detailed description of the 24 scenarios is listed in “Additional file 1”.

#### Data generating process

We first generated the random variable *Z* following the Beta (6, 2) distribution for 1,500 subjects. Then we created the exposure variable *X* for each subject based on the subject-specific probability of exposure. Finally, the outcome conditional on the exposure status and the covariate was randomly generated with the log-binomial distribution. For each of the 24 scenarios described above, the values of *α*_0_ and *β*_0_ were searched iteratively among all the possible values to guarantee that the expose and the outcome rates reached the designed level (50% for exposure and 10%, 25% or 40% for outcome). The *α*_0_ and *β*_0_ for each scenario can be found in Additional file 1. To study the differences between the two models in samples of moderate sizes, the same process was repeated to generate another set of data with n = 500.

#### Contaminating the datasets

For each of the 24 scenarios, the simulation datasets were contaminated by flipping the outcomes (*Y* becomes 1-*Y*) of the records that had the extreme probabilities of having *Y* = 1 (i.e., either very low or very high *p*_*y*_). Models were assessed by using the original simulated datasets and the datasets contaminated at the following level.

Contamination rates:

0% – original datasets

2% – flipping the outcomes of records with *p*_*y*_ at bottom or top 1%

5% – flipping the outcomes of records with *p*_*y*_ at bottom or top 2.5%

Although it is not realistic to expect outliers to solely come from observations with very low or very high probabilities of having the outcome, outliers resulting from flipping the outcome are possible due to documentation or data entry errors. For example, “0” (“not having the disease”) may be erroneously entered into the study database as “1” (“the disease was present”). Compared to adding outliers from a distribution that is different from the underlying one, the flipping approach produced outliers that are more likely to be leverage points. In other words, they are more likely to impact the estimates.

### Measures of model performance

For each scenario, the simulation process was repeated 1,000 times to estimate the relative bias, standard error and mean square error (MSE) for the log-binomial model and the robust Poisson model. The relative bias was calculated as the average of the 1,000 estimated RR in log scale minus the log of the true RR divided by the log of the true RR. Standard error was defined as the empirical standard error of the estimated RR in log scale over all 1,000 simulations. MSE was calculated by taking the sum of the squared bias in log scale and the variances.

Due to the non-convergence issue in standard statistical software, we used the COPY method to generate the estimates of the log-binomial models. However, the accuracy of parameter estimates depends on the number of virtual copies. For this evaluation the number of copies were set to 1,000,000, the default of the COPY method [17]. All the datasets were generated and analyzed using SAS Version 9.2 [18].

## Results

### Relative bias

**Figure 1:**
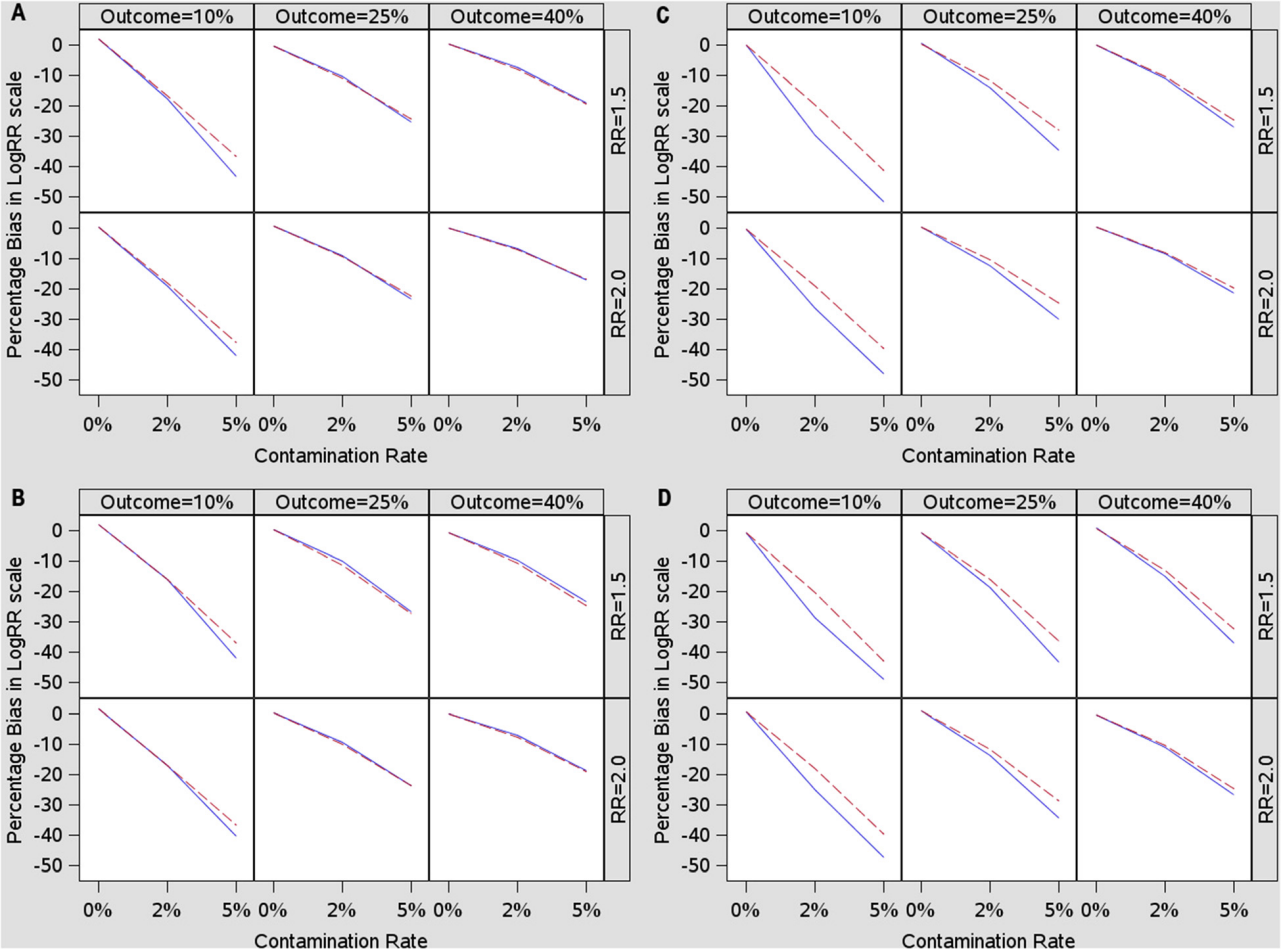
**Relative bias (%) in log scale (n = 1500). A**. Association between Z and Y: Linear; Level of association between Z and X, Z and Y: Moderate. **B**. Association between Z and Y: Linear; Level of association between Z and X, Z and Y: Strong. **C**. Association between Z and Y: Non-Linear; Level of association between Z and X, Z and Y: Moderate. **D**. Association between Z and Y: Non-Linear; Level of association between Z and X, Z and Y: Strong. Robust Poisson: Red dotted lines. Log-Binomial: Blue solid lines.

Table 1 and Figure 1 revealed the relative biases of the estimated RR in log scale from the two models in each of the 24 scenarios mentioned above when n = 1,500. As expected, both models accurately estimated *β*_1_ or log (*RR*) when the simulated databases were not contaminated (level of contamination = 0%). When the data were contaminated (level of contamination > 0%), the relative biases were all negative, indicating that the point estimates were biased towards null. For a fixed RR and an outcome rate, the absolute value of the relative biases increased quickly with the level of contamination. However, the pace of such an increase varied by the outcome rate. Scenarios with lower outcome rates seemed to be associated with more elevated absolute relative biases.

**Table 1:** Relative bias (%) in log scale (n = 1500)

**RR**	**Prob (Y = 1)**	**Contamination rate**	**Association bet Z and Y: Linear**		**Association bet Z and Y: Linear**		**Association bet Z and Y: Non-Linear**		**Association bet Z and Y: Non-Linear**	
			**Level of association bet Z and X, Z and Y: Moderate**		**Level of association bet Z and X, Z and Y: Strong**		**Level of association bet Z and X, Z and Y: Moderate**		**Level of association bet Z and X, Z and Y: Strong**	
			**LB**	**RP**	**LB**	**RP**	**LB**	**RP**	**LB**	**RP**
1.5	10%	0%	1.8	1.8	2.0	2.0	0.0	0.0	−0.6	−0.8
		2%	−17.8	−16.7	−16.0	−16.0	−30.0	−19.5	−28.5	−20.4
		5%	−43.4	−36.7	−41.8	−36.9	−51.7	−41.3	−49.1	−43.0
	25%	0%	−0.4	−0.4	0.4	0.3	0.5	0.5	−0.8	−0.8
		2%	−10.4	−10.9	−10.1	−11.4	−14.1	−11.8	−18.6	−16.2
		5%	−25.3	−24.4	−26.6	−27.3	−34.6	−28.2	−43.4	−36.2
	40%	0%	0.2	0.2	−0.7	−0.7	−0.0	−0.1	0.9	0.7
		2%	−7.5	−8.0	−9.8	−10.8	−11.0	−10.5	−14.9	−13.1
		5%	−19.2	−19.4	−23.4	−24.6	−26.9	−24.7	−36.9	−32.2
2.0	10%	0%	0.4	0.4	1.5	1.5	−0.2	−0.2	0.6	0.7
		2%	−18.9	−18.1	−17.1	−16.8	−26.3	−19.0	−25.0	−18.0
		5%	−42.0	−37.5	−40.2	−36.7	−47.9	−39.5	−47.4	−39.6
	25%	0%	0.5	0.5	0.3	0.3	0.3	0.3	0.9	0.8
		2%	−9.0	−9.2	−9.2	−9.9	−12.3	−10.4	−13.7	−11.7
		5%	−23.3	−22.2	−23.7	−23.6	−30.0	−24.7	−34.5	−28.5
	40%	0%	−0.1	−0.1	0.1	0.1	0.3	0.3	−0.3	−0.3
		2%	−6.7	−7.0	−7.2	−7.8	−8.4	−8.0	−10.9	−10.2
		5%	−16.9	−16.6	−18.5	−19.0	−21.2	−19.5	−26.6	−24.5

Association between Z and X always linear.

LB: Log-Binomial Model.

RP: Robust Poisson.

Relative bias was defined as the average of the 1,000 estimated RR in log scale minus the log of the true RR divided by the log of the true RR.

#### Models with linear confounder Z

When the models contained a linear confounder Z, the two models yielded comparable relative biases (Table 1, Figures 1A and B) except for a few scenarios in which the biases from the log-binomial models were slightly larger than those of the robust Poisson models. The largest differences occurred when the contamination rate was 5% and the outcome rate was 10%, in which the log-binomial models had 3.5%-6.7% higher biases compared to the corresponding robust Poisson models.

#### Models with non-linear confounder Z

With the non-linear confounder Z, the robust Poisson model outperformed the log-binomial model when the data was contaminated (Table 1, Figure 1C and D). The difference between the two models was visible even when the level of contamination was only 2%. The magnitude of difference between the two models also varied with the outcome rate. A larger difference was associated with smaller outcomes.

### Standard Error (SE)

When the simulated databases were not contaminated (level of contamination = 0%), the SEs of the two models were identical at the 2nd decimal point (Table 2). When the levels of contamination were greater than 0%, the estimated SEs remained comparable between the two models when the outcome rates were high (25% or 40%). However, when the outcome rate was low (10%), the SEs derived from the log-binomial models seemed to be slightly higher than those of robust Poisson in some of the scenarios.

**Table 2:** Standard error in log scale (n = 1500)

**RR**	**Prob (Y = 1)**	**Contamination rate**	**Association bet Z and Y: Linear**		**Association bet Z and Y: Linear**		**Association bet Z and Y: Non-Linear**		**Association bet Z and Y: Non-Linear**	
			**Level of association bet Z and X, Z and Y: Moderate**		**Level of association bet Z and X, Z and Y: Strong**		**Level of association bet Z and X, Z and Y: Moderate**		**Level of association bet Z and X, Z and Y: Strong**	
			**LB**	**RP**	**LB**	**RP**	**LB**	**RP**	**LB**	**RP**
1.5	10%	0%	0.16	0.16	0.16	0.16	0.16	0.16	0.16	0.16
		2%	0.14	0.14	0.14	0.14	0.15	0.14	0.14	0.14
		5%	0.13	0.12	0.13	0.12	0.15	0.13	0.15	0.13
	25%	0%	0.10	0.10	0.09	0.09	0.09	0.09	0.09	0.09
		2%	0.09	0.09	0.09	0.09	0.09	0.09	0.09	0.09
		5%	0.08	0.08	0.08	0.08	0.09	0.09	0.08	0.08
	40%	0%	0.06	0.06	0.06	0.07	0.06	0.06	0.06	0.06
		2%	0.06	0.06	0.06	0.06	0.06	0.06	0.06	0.06
		5%	0.06	0.06	0.06	0.06	0.06	0.06	0.06	0.06
2.0	10%	0%	0.17	0.17	0.16	0.16	0.16	0.16	0.17	0.17
		2%	0.15	0.15	0.15	0.15	0.16	0.15	0.15	0.15
		5%	0.14	0.13	0.13	0.13	0.15	0.13	0.16	0.13
	25%	0%	0.10	0.10	0.10	0.10	0.10	0.10	0.10	0.10
		2%	0.09	0.09	0.10	0.10	0.09	0.09	0.09	0.09
		5%	0.09	0.09	0.09	0.09	0.09	0.08	0.08	0.09
	40%	0%	0.07	0.07	0.07	0.07	0.07	0.07	0.07	0.07
		2%	0.07	0.07	0.07	0.07	0.07	0.07	0.07	0.07
		5%	0.07	0.07	0.07	0.07	0.07	0.07	0.07	0.07

Association between Z and X always linear.

LB: Log-Binomial Model.

RP: Robust Poisson.

Standard error was defined as the empirical standard error of the estimated RR in log scale over all 1,000 simulations.

### Mean Square Error (MSE)

The same pattern was observed for the MSEs as those of relative biases (Table 3). When the models contained a linear confounder Z, the two models had comparable MSEs except when the outcome rate was 10% and the contamination reached 5%. When the models contained a non-linear confounder Z, the two models diverged even when the degree of contamination was only 2% (Table 3).

**Table 3:** Mean Square Error (MSE) in log scale (n = 1500)

**RR**	**Prob (Y = 1)**	**Contamination rate**	**Association bet Z and Y: Linear**		**Association bet Z and Y: Linear**		**Association bet Z and Y: Non-Linear**		**Association bet Z and Y: Non-Linear**	
			**Level of association bet Z and X, Z and Y: Moderate**		**Level of association bet Z and X, Z and Y: Strong**		**Level of association bet Z and X, Z and Y: Moderate**		**Level of association bet Z and X, Z and Y: Strong**	
			**LB**	**RP**	**LB**	**RP**	**LB**	**RP**	**LB**	**RP**
1.5	10%	0%	0.025	0.025	0.027	0.027	0.025	0.025	0.024	0.024
		2%	0.026	0.025	0.025	0.024	0.036	0.026	0.033	0.026
		5%	0.049	0.038	0.045	0.038	0.066	0.044	0.063	0.046
	25%	0%	0.008	0.008	0.008	0.008	0.009	0.009	0.008	0.008
		2%	0.010	0.010	0.009	0.010	0.011	0.010	0.013	0.012
		5%	0.018	0.017	0.019	0.019	0.027	0.020	0.038	0.028
	40%	0%	0.004	0.004	0.004	0.004	0.004	0.004	0.004	0.004
		2%	0.005	0.005	0.006	0.006	0.006	0.006	0.008	0.007
		5%	0.010	0.010	0.013	0.014	0.016	0.014	0.027	0.021
2.0	10%	0%	0.028	0.028	0.027	0.027	0.027	0.027	0.029	0.029
		2%	0.040	0.038	0.035	0.035	0.057	0.039	0.053	0.038
		5%	0.105	0.085	0.096	0.081	0.134	0.091	0.135	0.093
	25%	0%	0.009	0.009	0.010	0.010	0.009	0.009	0.009	0.010
		2%	0.012	0.013	0.014	0.014	0.016	0.014	0.018	0.016
		5%	0.034	0.031	0.013	0.035	0.051	0.036	0.065	0.047
	40%	0%	0.005	0.005	0.005	0.005	0.005	0.005	0.005	0.005
		2%	0.007	0.007	0.007	0.008	0.008	0.008	0.011	0.010
		5%	0.018	0.018	0.021	0.022	0.026	0.023	0.039	0.034

Association between Z and X always linear.

LB: Log-Binomial Model.

RP: Robust Poisson.

Mean square error was calculated by taking the sum of the squared bias in log scale and the variances.

When the simulation was conducted based on samples of moderate sizes (n = 500), the same pattern was observed. As expected, the SEs based on the small samples were larger compared to those derived from large samples (n = 1,500). The results summarized from samples of size 500 were included in “Additional file 2”.

## Discussion

In this study, evaluation was performed on the statistical properties of the two most popular model-based approaches to estimate RR for common binary outcomes in various scenarios when outliers existed. The results suggest that for data coming from a population in which the true relationship between the outcome and the covariate is not in a simple form (e.g., containing a higher order term), the robust Poisson model consistently outperforms the log-binomial model even when the level of contamination is low (e.g., 2%). Statistical software utilizes iterative weighted least squares (IWLS) approach or variations of IWLS to find MLEs for generalized linear models. For log-binomial models, the weights used by the IWLS approach contain the term 1/(1-*p*), where *p* = exp (*X*^*T*^*β*) with a range from 0 to 1 [19]. Lumley et al. pointed out that the MLE of a log-binomial model is likely to be too sensitive to outliers because a very large *p* (referred to as *μ* by the authors) has a large influence on the weights, even though the sum of the covariate values are still bounded [15]. In our study both the MLE, generated by the log-binomial models and the pseudo-likelihood estimators, produced by the robust Poisson models, were deteriorated when outliers were introduced. However, the level of deterioration differed when the relationships between the confounder and the outcome was not in a simple form, possibly due to the bigger “*μ*”s yielded by the log-binomial model when the higher-order term of *Z* was added into the model.

Deddens and Pertersen compared the log-binomial and robust Poisson models by using three real-life examples [20]. Out of the three examples, two produced different point estimates and SEs. In one of these two examples, the difference was nearly two folds for both point estimates and SEs for one of the covariates. The authors concluded that “the decision on which method to use is very important” [20]; however, since the truth was unknown, it is unlikely to tell that between the log-binomial model and the robust Poisson model, which one can yield estimates that are closer to the truth. In one of the two examples in which differences between the two models were observed, the model contained a higher-order (quadratic) term. It is unclear whether or not the complexity of the model degenerated the performance of the models, especially for the log-binomial model.

Of the two methods, the log-binomial method is generally preferred due to the fact that the MLEs estimated by the log-binomial models are more efficient compared to the pseudo-likelihood estimators used by the robust Poisson models ( [10], page 2300). Spiegelman and Hertzmark recommend using the log-binomial models over the robust Poisson models when convergence is not an issue [21]. Very small differences were observed in a simulation study with a sample size of 100 and a single independent variable with a uniform distribution [14]. When data perfectly follows a log-binomial distribution (i.e., without outliers), the current study did not observe any difference in either biases or variances between the log-binomial and the robust Poisson models for large (n = 1,500) and moderate (n = 500) sample sizes. It appears that the gain in efficiency is beneficial to log-binomial models only for samples of small sizes.

It is not a surprise to observe negative biases when the simulation datasets were contaminated, because flipping the outcomes of the records that have the very low or very high probabilities tend to weaken the associations between the exposure and the outcome leading the associations towards null. The observation of more elevated biases when outcome rate = 10% compared to those of 25% and 40% comes with no surprise either since the impact of flipping on the estimates is expected to be more significant for scenarios with more extreme outcomes (close to 0% or 100%).

Robust methods to detect outliers especially high leverage points for logistic regression models are available in popular statistical software packages [22-24]. However, similar approaches for log-binomial models are not yet available in commercial software packages although the adoption of the diagnostic statistics from those of logistic regression models were demonstrated and applied in a real life example [25]. Efforts to develop goodness of fit tests resulted in reasonable type I errors yet low to moderate power [25]. For this reason, the robust Poisson model seems to be a more attractive choice due to its capability of providing more robust results when outliers are undetected.

For the COPY method, the accuracy of parameter estimates depends on the number of virtual copies. Peterson and Deddens pointed out that “with 10,000 copies the results were generally accurate to three decimal places” in their scenarios [17]. Therefore, the number of virtual copies we used (1,000,000) should provide accuracies that are high enough for our evaluation. The number of virtual copies should be carefully chosen, because a high number of virtual copies may result in failure of convergence.

Occasionally, failure of convergence remains to be an issue for log-binomial models even if the COPY method is applied. Peterson and Deddens [14] included a continuous exposure variable (referred to as the continuous covariate by the authors) when applying the COPY method in the simulation and reported a range of convergence between 70% and 100%. In this study where C, the number of the virtue copies, was set to be 1,000,000, the COPY method converged in all 1,000 simulations in all the 48 scenarios with the linear confounder, and in 30 of out 48 scenarios with the non-linear confounder. In the 18 scenarios in which the COPY method did not converge in all 1,000 simulations (failed in 1 or more simulations), the converge rates ranged from 0.983 to 0.999 (median 0.996). If the COPY method fails to converge and the maximum likelihood-based estimators are desired, one can choose the Non-Linear Programming (NLP) procedure in SAS [26]. The NLP method is computationally expensive. However, it does not encounter any convergence issues.

Given the lack of robustness of log-binomial models, the authors recommend using robust Poisson models to estimate RR when there are continuous covariates in the model, especially when the covariates are not in a simple linear form. Due to the concern of lack of efficiency for the robust Poisson models for small samples, log-binomial may still be the choice when the sample size is small.

A potential limitation of this study is that complex forms between the confounder and the outcome were generated by a quadratic equation. It is not clear whether or not the findings can be generalized to other complex situations. In addition, all of the outliers generated occurred to records with very low or very high probabilities and such outliers are more likely to be leverage points. The impact of the outliers generated by this study could be more significant compared to that of another study with outliers that were differently distributed.

In summary, the current study revealed the evidence to support the robustness of the robust Poisson model in various scenarios. Further research should focus on the model misspecification due to deviations of underlying probabilities. It is desirable for future studies to develop methods to identify leverage points and efficient goodness-of-fit test for log-binomial models.

## Conclusions

The robust Poisson models are more robust to outliers compared to the log-binomial models when estimating relative risks or risk ratios for common binary outcomes. Users should be aware of the limitations when choosing appropriate models to estimate relative risks or risk ratios.

## Abbreviations

OR: Odds ratio; RR: Relative risk; MLE: Maximum likelihood estimator; GEE: Generalized estimation equation; SE: Standard error; MSE: Mean square error.

## Competing interests

The authors declare that they have no competing interests.

## Authors’ contributions

WC conceived and carried out the study, and drafted the manuscript. JS participated in the design, data generation and interpretation of the analyses. LQ participated in the design, simulation and interpretation of the analyses. SA participated in the design and provided guidance. All the authors read and approved the final manuscript.

## Pre-publication history

The pre-publication history for this paper can be accessed here:

http://www.biomedcentral.com/1471-2288/14/82/prepub

## Supplementary Material

Additional file 1 Description of 24 Scenarios for Data Generation. Click here for file

Additional file 2 Relative Bias (%), Standard Error (SE) and Mean Square Error (MSE) When n = 500. Click here for file
