# Supplementary material for: Comparison of robustness to outliers between robust poisson models and log-binomial models when estimating relative risks for common binary outcomes: a simulation study
Source: BMC Med Res Methodol. 2014 Jun 26;14:82. doi: 10.1186/1471-2288-14-82 (PMC4079617; doi:10.1186/1471-2288-14-82)
Supplement: Additional file 1 — Description of 24 Scenarios for Data Generation. [file 1471-2288-14-82-S1.pdf]

Description of 24 Scenarios for Data Generation

β_1= log(RR)	P(Y=1)	Association between Z and Y: Linear[1]				Association between Z and Y: Non-Linear[2]			
		Level of association between Z and X, Z and Y				Level of association between Z and X, Z and Y			
		Moderate: α_1= β_2=log(2)		Strong: α_1= β_2=log(4)		Moderate: α_1= β_2=log(2)		Strong: α_1= β_2=log(4)	
		α_0	β_0	α_0	β_0	α_0	β_0	α_0	β_0
	10%		-3.0518		-3.5885		-3.2636		-4.0302
log(1.5)	25%		-2.1349		-2.6714		-2.3473		-3.1136
	40%	-0.5188	-1.6649	-1.0425	-2.2016	-0.5188	-1.8769	-1.0425	-2.6440
	10%		-3.2346		-3.7722		-3.4472		-4.2161
log(2.0)	25%		-2.3191		-2.8561		-2.5300		-3.3006
	40%		-1.8485		-2.3863		-2.0618		-2.8298

X is a binary treatment/exposure variable ($X = 1$ for treatment/exposure and $X = 0$ for non-treatment/non-exposure) from a binomial distribution with the probability of $X = 1$ fixed at 50%. Y is a binary common outcome from a population with the probability of $Y = 1$ varying from 10%, 25% to 40%.

$Z \sim \text{Beta}(6, 2)$

For all scenarios: $\text{logit}(p_x) = \alpha_0 + \alpha_1 Z$

[1]Linear confounder Z: $\log(p_y) = \beta_0 + \beta_1 X + \beta_2 Z$

[2]Non-linear confounder Z: $\log(p_y) = \beta_0 + \beta_1 X + \beta_2 Z + (0.5 \times \beta_2) Z^2$
