# Supplementary material for: Comparison of robustness to outliers between robust poisson models and log-binomial models when estimating relative risks for common binary outcomes: a simulation study
Source: BMC Med Res Methodol. 2014 Jun 26;14:82. doi: 10.1186/1471-2288-14-82 (PMC4079617; doi:10.1186/1471-2288-14-82)
Supplement: Additional file 2 — Relative Bias (%), Standard Error (SE) and Mean Square Error (MSE) When n = 500. [file 1471-2288-14-82-S2.pdf]

Table A1. Relative Bias (%) in Log Scale When n=500

RR	Prob (Y=1)	Contamination Rate	Association bet Z and Y: Linear<br><br>Level of association bet Z and X, Z and Y: Moderate		Association bet Z and Y: Linear<br><br>Level of association bet Z and X, Z and Y: Strong		Association bet Z and Y: Non-Linear<br><br>Level of association bet Z and X, Z and Y: Moderate		Association bet Z and Y: Non-Linear<br><br>Level of association bet Z and X, Z and Y: Strong	
			LB	PR	LB	RP	LB	RP	LB	RP
1.5	10%	0%<br>2%<br>5%	3.1<br>-17.3<br>-43.0	3.2<br>-15.5<br>-35.3	4.4<br>-14.2<br>-40.6	4.4<br>-13.8<br>-35.1	0.4<br>-28.1<br>-51.8	0.4<br>-17.8<br>-39.7	0.1<br>-24.7<br>-46.8	-0.1<br>-17.2<br>-39.0
	25%	0%<br>2%<br>5%	0.0<br>-9.9<br>-25.4	-0.4<br>-10.3<br>-24.2	1.3<br>-9.5<br>-27.0	1.2<br>-10.7<br>-27.3	3.4<br>-11.3<br>-32.2	3.4<br>-8.8<br>-25.3	-0.5<br>-18.1<br>-43.6	-0.6<br>-15.4<br>-35.9
	40%	0%<br>2%<br>5%	0.2<br>-7.9<br>-19.7	0.1<br>-8.2<br>-19.6	0.2<br>-8.8<br>-22.4	0.2<br>-10.0<br>-23.5	1.0<br>-9.6<br>-26.1	0.8<br>-9.1<br>-23.7	1.4<br>-14.2<br>-36.0	1.2<br>-12.2<br>-31.2
2.0	10%	0%<br>2%<br>5%	2.1<br>-17.7<br>-40.4	2.1<br>-16.6<br>-35.6	4.0<br>-15.6<br>-39.7	4.0<br>-15.0<br>-35.3	2.4<br>-21.5<br>-43.8	2.4<br>-14.4<br>-35.5	2.6<br>-22.0<br>-45.5	2.7<br>-15.4<br>-37.0
	25%	0%<br>2%<br>5%	0.1<br>-9.7<br>-24.0	0.1<br>-9.8<br>-22.6	0.3<br>-9.2<br>-23.8	0.2<br>-9.7<br>-23.4	0.3<br>-12.2<br>-29.4	0.3<br>-10.1<br>-24.0	2.0<br>-12.3<br>-33.1	1.8<br>-10.0<br>-26.9
	40%	0%<br>2%<br>5%	-0.9<br>-7.7<br>-17.8	-0.9<br>-7.9<br>-17.5	0.6<br>-6.7<br>-18.2	0.5<br>-7.3<br>-18.6	0.2<br>-8.3<br>-21.0	0.2<br>-7.7<br>-19.1	-0.3<br>-11.0<br>-26.7	-0.1<br>-10.0<br>-24.1

Association between Z and X always linear
LB: Log-Binomial Model
RP: Robust Poisson
Relative bias was defined as the average of the 1,000 estimated RR in log scale minus the log of the true RR divided by the log of the true RR.

Table A2. Standard Error in Log Scale When n=500

RR	Prob (Y=1)	Contamination Rate	Association bet Z and Y: Linear<br><br>Level of association bet Z and X, Z and Y: Moderate		Association bet Z and Y: Linear<br><br>Level of association bet Z and X, Z and Y: Strong		Association bet Z and Y: Non-Linear<br><br>Level of association bet Z and X, Z and Y: Moderate		Association bet Z and Y: Non-Linear<br><br>Level of association bet Z and X, Z and Y: Strong	
			LB	PR	LB	RP	LB	RP	LB	RP
1.5	10%	0%<br>2%<br>5%	0.27<br>0.24<br>0.23	0.27<br>0.24<br>0.21	0.29<br>0.25<br>0.23	0.29<br>0.25<br>0.22	0.29<br>0.26<br>0.26	0.29<br>0.26<br>0.23	0.28<br>0.25<br>0.24	0.28<br>0.25<br>0.22
	25%	0%<br>2%<br>5%	0.17<br>0.17<br>0.16	0.17<br>0.17<br>0.16	0.16<br>0.16<br>0.15	0.16<br>0.16<br>0.15	0.17<br>0.16<br>0.16	0.17<br>0.16<br>0.15	0.16<br>0.16<br>0.15	0.16<br>0.16<br>0.15
	40%	0%<br>2%<br>5%	0.11<br>0.11<br>0.11	0.11<br>0.11<br>0.11	0.11<br>0.11<br>0.11	0.11<br>0.11<br>0.11	0.11<br>0.11<br>0.11	0.11<br>0.11<br>0.10	0.11<br>0.11<br>0.11	0.11<br>0.11<br>0.11
2.0	10%	0%<br>2%<br>5%	0.30<br>0.27<br>0.24	0.30<br>0.26<br>0.23	0.30<br>0.26<br>0.24	0.30<br>0.25<br>0.22	0.30<br>0.27<br>0.25	0.30<br>0.26<br>0.23	0.30<br>0.26<br>0.25	0.30<br>0.26<br>0.23
	25%	0%<br>2%<br>5%	0.17<br>0.16<br>0.16	0.17<br>0.16<br>0.15	0.17<br>0.17<br>0.16	0.17<br>0.17<br>0.16	0.17<br>0.17<br>0.16	0.17<br>0.16<br>0.15	0.17<br>0.17<br>0.16	0.18<br>0.17<br>0.16
	40%	0%<br>2%<br>5%	0.12<br>0.12<br>0.11	0.12<br>0.12<br>0.11	0.12<br>0.11<br>0.11	0.12<br>0.12<br>0.11	0.12<br>0.12<br>0.12	0.12<br>0.12<br>0.11	0.12<br>0.12<br>0.12	0.12<br>0.12<br>0.12

Association between Z and X always linear
LB: Log-Binomial Model
RP: Robust Poisson
Standard error was defined as the empirical standard error of the estimated RR in log scale over all 1,000 simulations.

Table A3. Mean Square Error (MSE) in Log Scale When n=500

RR	Prob (Y=1)	Contamination Rate	Association bet Z and Y: Linear<br><br>Level of association bet Z and X, Z and Y: Moderate		Association bet Z and Y: Linear<br><br>Level of association bet Z and X, Z and Y: Strong		Association bet Z and Y: Non-Linear<br><br>Level of association bet Z and X, Z and Y: Moderate		Association bet Z and Y: Non-Linear<br><br>Level of association bet Z and X, Z and Y: Strong	
			LB	PR	LB	RP	LB	RP	LB	RP
1.5	10%	0%<br>2%<br>5%	0.073<br>0.063<br>0.083	0.073<br>0.061<br>0.065	0.082<br>0.066<br>0.079	0.082<br>0.066<br>0.068	0.083<br>0.082<br>0.111	0.083<br>0.072<br>0.079	0.077<br>0.072<br>0.094	0.077<br>0.067<br>0.074
	25%	0%<br>2%<br>5%	0.028<br>0.029<br>0.035	0.028<br>0.029<br>0.034	0.027<br>0.026<br>0.034	0.027<br>0.027<br>0.034	0.028<br>0.028<br>0.042	0.028<br>0.027<br>0.034	0.026<br>0.030<br>0.053	0.026<br>0.028<br>0.043
	40%	0%<br>2%<br>5%	0.013<br>0.013<br>0.018	0.013<br>0.013<br>0.018	0.012<br>0.013<br>0.020	0.012<br>0.014<br>0.021	0.012<br>0.013<br>0.022	0.012<br>0.013<br>0.020	0.013<br>0.016<br>0.034	0.013<br>0.015<br>0.028
2.0	10%	0%<br>2%<br>5%	0.087<br>0.088<br>0.138	0.087<br>0.083<br>0.113	0.089<br>0.077<br>0.131	0.089<br>0.075<br>0.110	0.092<br>0.098<br>0.156	0.092<br>0.082<br>0.113	0.091<br>0.092<br>0.164	0.091<br>0.079<br>0.119
	25%	0%<br>2%<br>5%	0.029<br>0.031<br>0.053	0.029<br>0.031<br>0.049	0.029<br>0.032<br>0.052	0.030<br>0.032<br>0.051	0.029<br>0.034<br>0.066	0.029<br>0.031<br>0.051	0.031<br>0.036<br>0.079	0.031<br>0.034<br>0.061
	40%	0%<br>2%<br>5%	0.015<br>0.017<br>0.028	0.015<br>0.017<br>0.027	0.014<br>0.015<br>0.028	0.014<br>0.016<br>0.029	0.015<br>0.017<br>0.035	0.015<br>0.017<br>0.031	0.014<br>0.020<br>0.048	0.014<br>0.019<br>0.041

Association between Z and X always linear
LB: Log-Binomial Model
RP: Robust Poisson
Mean square error (MSE) was calculated by taking the sum of the squared bias in log scale and the variances.
